# Superior efficacy of co-treatment with dual PI3K/mTOR inhibitor NVP-BEZ235 and pan-histone deacetylase inhibitor against human pancreatic cancer

**DOI:** 10.18632/oncotarget.724

**Published:** 2012-11-15

**Authors:** Sreedhar Venkannagari, Warren Fiskus, Karissa Peth, Peter Atadja, Manuel Hidalgo, Anirban Maitra, Kapil N. Bhalla

**Affiliations:** ^1^ The University of Kansas Cancer Center, Kansas City, KS; ^2^ Novartis Institute for Biomedical Research Inc., Cambridge, MA; ^3^ Centro Nacional de Investigaciones Oncologicas, C Melchor Fernandez Almagro 3, Madrid, Spain; ^4^ The Sol Goldman Pancreatic Cancer Research Center, Johns Hopkins University, Baltimore, MD

**Keywords:** Pancreatic cancer, pan-HDAC inhibitor, BEZ235, mTOR, AKT

## Abstract

Genetic alterations activating K-RAS and PI3K/AKT signaling are also known to induce the activity of mTOR kinase through TORC1 and TORC2 complexes in human pancreatic ductal adenocarcinoma (PDAC). Here, we determined the effects of the dual PI3K and mTOR inhibitor, NVP-BEZ235 (BEZ235), and the pan-histone deacetylase inhibitor panobinostat (PS) against human PDAC cells. Treatment with BEZ235 or PS inhibited cell cycle progression with induction of the cell cycle inhibitory proteins, p21 waf1 and p27 kip1. BEZ235 and PS also dose dependently induced loss of cell viability of the cultured PDAC cells, associated with depletion of phosphorylated (p) AKT, as well as of the TORC1 substrates 4EBP1 and p70S6 kinase. While inhibiting p-AKT, treatment with PS induced the levels of the pro-apoptotic proteins BIM and BAK. Co-treatment with BEZ235 and PS synergistically induced apoptosis of the cultured PDAC cells. This was accompanied by marked attenuation of the levels of p-AKT and Bcl-x_L_ but induction of BIM. Although in vivo treatment with BEZ235 or PS reduced tumor growth, co-treatment with BEZ235 and PS was significantly more effective in controlling the xenograft growth of Panc1 PDAC cells in the nude mice. Furthermore, co-treatment with BEZ235 and PS more effectively blocked tumor growth of primary PDAC heterotransplants (possessing K-RAS mutation and AKT2 amplification) subcutaneously implanted in the nude mice than each agent alone. These findings demonstrate superior activity and support further in vivo evaluation of combined treatment with BEZ235 and PS against PDAC that possess heightened activity of RAS-RAF-ERK1/2 and PI3K-AKT-mTOR pathways.

## INTRODUCTION

Advanced exocrine pancreatic ductal adenocarcinoma (PDAC) is a lethal disease with a survival rate of less than 5% [1]. PDAC is a highly aneuploid cancer, associated with not only significant alterations in the copy numbers of several genes but also with a successive accumulation of gene mutations [2, 3]. Among the genes that are mutated in pancreatic cancer are *K-RAS* (90%), *TP53*/p53 (50-75%), *CDKN2A*/p16 (95%) and *SMAD4* (50%) [3]. In addition, *AKT2* (10-20%) amplification and mutations in *PTEN, BRAF, STK11* (LKB1) and *BRCA2* have been reported in a smaller percentage of PDAC [2, 3]. Activating mutation in *K-RAS* increases RAS-RAF-ERK1/2 activity, which is known to promote growth and survival of PDAC [2, 3]. K-RAS mutation may also cause aberrant activation of other intracellular signaling pathways, including the phosphatidylinositol-3-kinase (PI3K)-AKT/mammalian target of rapamycin (mTOR) signaling pathway [4]. In addition, activating mutations in PI3K or AKT2 amplification, or the loss of PTEN phosphatase activity, individually have been documented to augment PI3K-AKT-mTOR activity, which also promotes the growth and survival of PDAC [2-5]. Singly or in a combinatorial manner, these genetic alterations may contribute to the aggressive nature of the PDAC and confer resistance to the conventional and targeted agents [2, 6, 7].

AKT is a serine/threonine protein kinase, which is activated by phosphorylation at T308 by PI3K-PDK1 and at S473 residue by mTOR kinase associated with the TORC2 complex [8, 9]. AKT is known to phosphorylate FOXO3A, thereby inhibiting transcriptional activation of the pro-apoptotic proteins BIM and p27 [9, 10]. AKT also phosphorylates BAD, BIM and caspase-9, which leads to inhibition of apoptosis [8]. Through crosstalk with other signaling pathways, including, WNT, NFκB and MAPK, AKT activity also promotes tumor cell growth by up-regulating Myc and Cyclin D1 [4, 10]. AKT also activates the serine/threonine kinase activity of mTOR kinase, which is the active component of two multi-protein complexes, TORC1 and TORC2 [11, 12]. AKT also phosphorylates the proline-rich AKT substrate of 40 kDa (PRAS40) causing its detachment from the TORC1 complex, which it inhibits. Thus, AKT activates TORC1 in a PRAS40-dependent manner [4,11]. In addition, AKT-mediated phosphorylation also shuts down the GTPase activating protein (GAP) activity of TSC2-TSC1 for RHEB, whereby GTP-bound RHEB activates TORC1 [13]. Thus, AKT activity potentially activates TORC1 by two separate mechanisms. TORC1 directly phosphorylates the eukaryotic translational initiation factor 4E (eIF4E)-binding protein (4EBP1) and S6 kinase1 (S6K1), which promotes protein synthesis in PDAC cells [9, 11, 13]. TORC1-mediated phosphorylation of 4EBP1 inhibits its binding to eIF4E, thereby allowing eIF4E to participate in the formation of eIF4F complex. This complex enables cap-dependent protein translation of pro-growth (Myc and Cyclin D1) and pro-survival proteins (e.g., MCL-1 and Bcl-x_L_) [4, 14, 15]. Loss of 4EBP1 was shown to increase tumorigenesis due to p53 inactivation, whereas an increase in 4EBP1 activity inhibited tumors driven by co-expression of mutant KRAS and PI3K [16, 17]. This created a compelling rationale to use mTOR inhibitors such as rapamycin or related ‘rapalogs’ against PDAC [18, 19]. Rapamycin and ‘rapalogs’ inhibit mTOR by allosterically inhibiting TORC1 but not TORC2 [20, 21]. It is the TORC2 complex-associated mTOR that phosphorylates AKT on the S473 residue, promoting its activity, which is uninhibited by rapamycin [9, 11, 22]. In addition, rapamycin has been shown to only incompletely inhibit 4EBP1 phosphorylation, leaving the rapamycin-resistant TORC1 mediated phosphorylation of 4EBP1 un-inhibited [20, 22]. In addition, recent reports have shown that several negative feedback loops limit the therapeutic efficacy of ‘rapalogs’ [20, 22]. TORC1 either directly or through activation of S6K1 phosphorylates IRS1, which promotes its degradation, thereby reducing the growth factor signaling downstream of receptor tyrosine kinases (RTKs) mediated through the PI3K-PDK1-AKT axis [11]. Recently, TORC1 was also shown to directly phosphorylate and stabilize GRB10, which negatively regulates the growth factor signaling through RTKs and the PI3K-PDK1-AKT axis [23, 24]. Therefore, the activity of rapamycin and related ‘rapalogs’ is impaired by the negative feedback loop that activates AKT through TORC1 inhibition, which promotes growth and survival of PDAC [22, 25].

NVP-BEZ235 is an orally bioavailable imidazoquinoline derivative that binds to the ATP-binding clefts of the class I PI3K and mTOR kinase, leading to inhibition of PI3K, as well as attenuation of TORC1 and TORC2 activity [26, 27]. By inhibiting PI3K, and TORC2, treatment with BEZ235 abrogates the feedback activation of AKT, as well as mediates more complete inhibition of 4EBP1 phosphorylation [28]. Based on this, BEZ235 has been documented to be active against transformed cells with increased activity of PI3K-AKT-TORC1, which results due to PIK3CA mutation, PIK3R1 mutation, PTEN loss, RTK dependent activation or AKT amplification [10, 28]. BEZ235 has been shown to exert G_0_/G_1_ cell cycle arrest and anti-proliferative responses in a variety of cancer cell types [26-30]. Recently, it was reported that treatment of non-small cell lung carcinoma (NSCLC) with BEZ235 resulted in regression of PIK3CA-mutated but not K-RAS-mutated tumors [31]. However, combined administration of BEZ235 with MEK1 inhibitor resulted in significant regression of K-RAS-mutated NSCLC tumors [31]. Treatment with pan-histone deacetylase inhibitors such as panobinostat (PS) has been documented to induce hyperacetylation and inhibition of the chaperone function of hsp90 [32]. This was shown to cause the misfolding, polyubiquitylation and degradation of hsp90-chaperoned client proteins, e.g., c-RAF, which inhibits the downstream signaling through MEK-ERK1/2 [32, 33]. In addition, HDAC inhibitor treatment leads to inhibition of cell cycle progression, as well as lowers the threshold for apoptosis by inducing pro-death, e.g. BAX, BAK, and BIM, and attenuating pro-survival proteins, e.g., Bcl-x_L_, MCL-1 and XIAP, through multiple mechanisms [34-36]. PS has also been shown to exhibit in vitro and in vivo anti-PDAC activity [37]. Therefore, collectively based on these observations, in present studies, we determined the in vitro and in vivo effects of BEZ235 and PS against human PDAC cells expressing mutant K-RAS and exhibiting increased activity of PI3K-AKT-mTOR axis. Our findings show that as compared to treatment with each agent alone, co-treatment with BEZ235 and PS synergistically induces apoptosis of PDAC cells and exerts superior in vivo activity in PDAC cell xenografts in the nude mice.

## RESULTS

**Figure 1:**
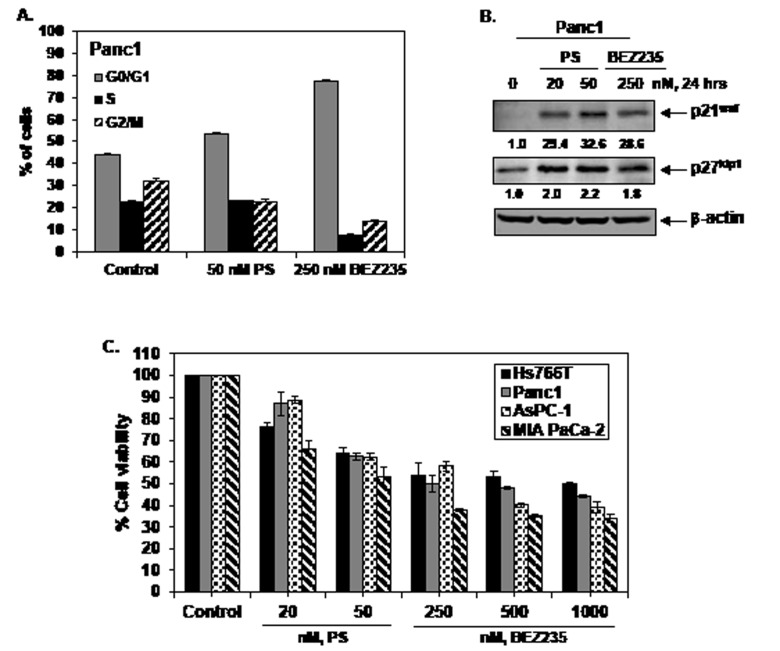
Treatment with PS or BEZ235 induces cell cycle growth arrest and reduces cell viability of cultured human pancreatic cancer cells A. Panc1 cells were treated with indicated concentrations of PS or BEZ235 for 24 hours. At the end of treatment, the cells were washed with 1X PBS and fixed in 70% ethanol. Cells were stained with propidium iodide and cell cycle status was determined by flow cytometry. Values represent the mean ± S.E.M. of three independent experiments. B. Panc1 cells were treated with the indicated concentrations of PS or BEZ235 for 24 hours. Then, total cell lysates were prepared and immunoblot analyses were performed for p21 and p27. The expression levels of β-actin in the lysates served as the loading control. C. Pancreatic cancer cells (Hs766T, Panc1, MIA PaCa-2 and AsPC-1) were treated with the indicated concentrations of PS and BEZ235 for 48 hours. Following this, the percentages of viable cells were assessed utilizing a Cell Counting Kit-8 (Dojindo Molecular Technologies). Columns represent the mean percentage of viable cells relative to the untreated controls; Bars represent the standard error of the mean.

Treatment with BEZ235 and PS inhibits cell cycle progression and mediates loss of cell viability in pancreatic cancer cells. We first determined the cell cycle effects of BEZ235 and PS in the cultured PDAC Panc1 cells. As shown in Figure 1A, exposure to 250 nM of BEZ235 for 24 hours markedly increased the accumulation of Panc1 cells in the G0/G1 phase with decline in the % of cells in the S and G2/M phases of the cell cycle. No further increase in this effect on cell cycle was observed at higher concentrations of BEZ235 (data not shown). Treatment with PS (50 nM for 24 hours) also increased the % of cells in G0/G1 phase and mediated a decline in the G2/M phase of the cell cycle (Figure 1A). The effects of PS and BEZ235 on the cell cycle were associated with a marked increase in the levels of the cell cycle inhibitory protein p21^waf^ but a more modest increase in p27^kip1^ levels in Panc1 cells (Figure 1B). Similar findings were also observed in Hs766T cells (data not shown). Figure 1C shows that a 48 hour exposure to PS or BEZ235 dose-dependently reduced the cell viability of each of the four cultured PDAC cell types, with the greatest effect seen in MIA PaCa-2 and the least effect in Hs766T cells.

**Figure 2:**
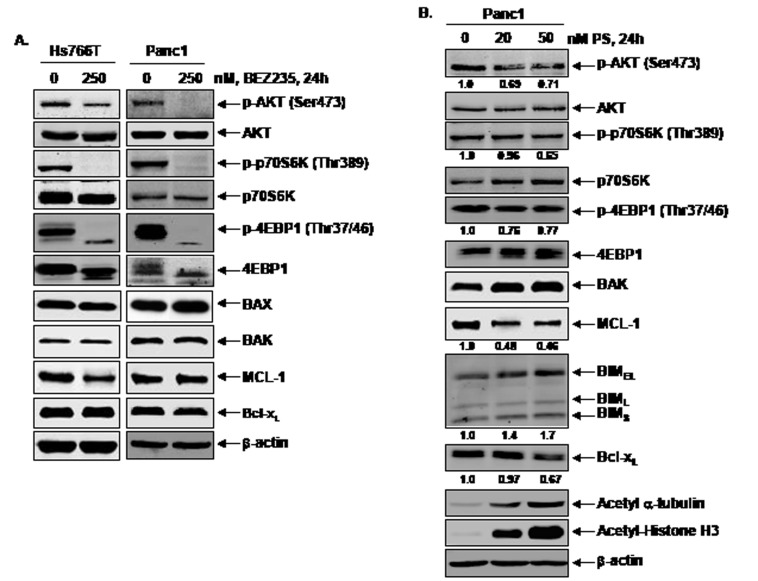
Effects of treatment with BEZ235 or PS on mTOR signaling and BCL2 family proteins in pancreatic cancer cells A. Hs766T and Panc1 cells were treated with the indicated concentrations of BEZ235 for 24 hours. Following this, total cell lysates were prepared and immunoblot analyses were performed for p-AKT (Ser473), AKT, p-p70S6K (Thr389), p70S6K, p-4EBP1 (Thr37/46), 4EBP1, BAX, BAK, MCL-1 and Bcl-x_L_. The expression levels of β-actin in the lysates served as the loading control. B. Panc1 cells were treated with the indicated concentrations of PS for 24 hours. After treatment, total cell lysates were prepared and immunoblot analyses were performed for p-AKT (Ser473), AKT, p-p70S6K (Thr389), p70S6K, p-4EBP1(Thr37/46), 4EBP1, BAK, MCL-1, BIM, Bcl-x_L_, acetyl α-tubulin and acetyl Histone H3. The expression levels of β-actin in the lysates served as the loading control. The numbers beneath the bands represent densitometry performed on representative immunoblots relative to the control cells.

**Figure 3:**
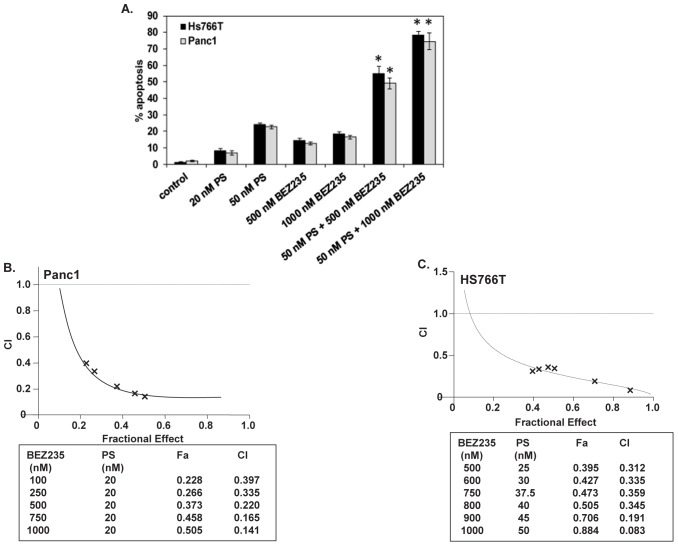
Co-treatment with BEZ235 and PS synergistically induces apoptosis of human pancreatic cancer cells A. Panc1 and Hs766T cells were treated with the indicated concentrations of PS and/or BEZ235 for 48 hours. Following this, the cells were trypsinized, washed with 1X PBS, and stained with annexin V and TO-PRO-3 iodide. The percentages of annexin V-positive, apoptotic cells were determined by flow cytometry. Columns represent the mean of three experiments; Bars, standard error of the mean. * indicates values significantly greater in the combination compared to treatment with either agent alone (p < 0.05 as determined by a two-tailed, paired t-test). B-C. Panc1 and Hs766T cells were treated with PS and/or BEZ235 for 48 hours. At the end of treatment, the cells were trypsinized, washed with 1X PBS, and stained with annexin V and TO-PRO-3 iodide. The percentages of annexin V-positive, apoptotic cells were determined by flow cytometry. Median dose effect and isobologram analyses were performed according to the methods of Chou and Talalay utilizing the commercially available software Calcusyn. The combination index (CI) values were calculated for 3 independent experiments. CI values less than 1.0 indicate the synergistic interaction of the two agents in the combination.

Effects of BEZ235 and PS treatment on PI3K/mTOR signaling in pancreatic cancer cells. We next determined the effects of treatment with BEZ235 on the key TORC1 and TORC2 substrates in Hs766T and Panc1 cells. Exposure to 250 nM of BEZ235 for 24 hours markedly depleted the levels of the TORC1 substrates p-4EBP1 (Thr37/46) and p-p70S6K (Thr389) and reduced the levels of the TORC2 substrate p-AKT (Ser473) (Figure 2A). Lower concentrations of BEZ235 mediated less pronounced effects on TORC1 and TORC2 substrates. There was also no significant effect of BEZ235 on the levels of BAX, BAK, or MCL-1 in the PDAC cells (Figure 2A). As previous studies have documented that treatment with PS reduces the levels of p-AKT (Ser473) and inhibits TORC1 activity in other tumor cell types [38], we next determined whether PS treatment also depleted TORC1 and TORC2 activity in PDAC cells. As previously noted in other cell types [32], exposure to PS caused hyperacetylation of both histone H3 and α-tubulin, inhibitory effects of PS on both class I (HDAC1, 2, & 3) and class IIB (HDAC6) HDACs (Figure 2B). Treatment with PS only modestly depleted the levels of p-4EBP1 (Thr37/46), p-p70S6K (Thr389) and p-AKT (Ser473) in Panc1 and Hs766T cells (Figure 2B and Supplemental Figure S2). PS treatment also induced BIM and BAK, but reduced the levels of Bcl-x_L_ and MCL-1, consistent with its ability to induce apoptosis in PDAC cells (Figure 2B & 3A and Supplemental Figure S2).

**Figure 4:**
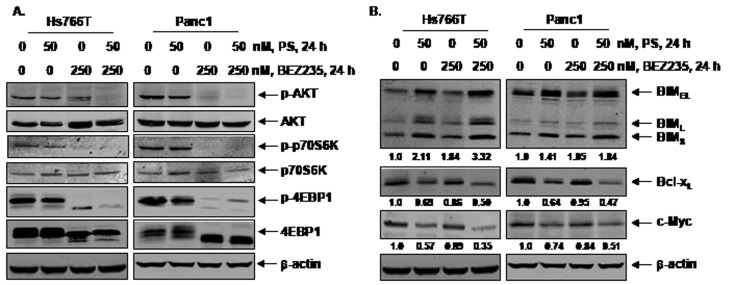
Co-treatment with BEZ235 enhances PS-mediated depletion of p-AKT, and Bcl-xL and induction of BIM in pancreatic cancer cells A.-B. Hs766T and Panc1 cells were treated with indicated concentrations of PS and/or BEZ235 for 24 hours. Following this, total cell lysates were prepared and immunoblot analyses were performed for p-AKT (Ser473), AKT, p-p70S6K (Thr389), p70S6K, p-4EBP1 (Thr37/46), 4EBP1, c-Myc, BIM, and Bcl-x_L_. The expression levels of β-actin in the lysates served as the loading control. The numbers beneath the bands represent densitometry performed on representative immunoblots relative to the control cells.

Co-treatment with PS and BEZ235 synergistically induces apoptosis of pancreatic cancer cells. We next determined the effects of combined treatment with BEZ235 and PS in Hs766T and Panc1 cells. Figure 3A shows that, compared to treatment with either agent alone, co-treatment with BEZ235 and PS induced significantly more apoptosis of Hs766T and Panc1 cells (p <0.05). Furthermore, median dose effect and isobologram analyses performed utilizing Calcusyn software showed that co-treatment with BEZ235 and PS synergistically induced apoptosis of Hs766T and Panc1 cells (Figure 3 B-C). We also compared the effects of combined treatment with BEZ235 and PS to treatment with each agent alone on AKT, TORC1 and TORC2 activity in the cultured PDAC cells. As shown in Figure 4A, in the PDAC cells, co-treatment with BEZ235 and PS was more active in depleting the levels of p-AKT (Ser473), p-p70S6K (Thr389), and p-4EBP1 (Thr37/46) than treatment with each agent alone. Next, we determined the effects of BEZ235 and PS on the levels of selective apoptosis regulators in PDAC cells. Figure 4B shows that co-treatment with BEZ235 and PS was more effective in inducing BIM_EL_, BIM_L_ and BIM_S_ in the PDAC cells. In addition, following treatment with the combination of BEZ235 and PS, a greater decline in Bcl-x_L_ and c-Myc levels was also observed (Figure 4B).

**Figure 5:**
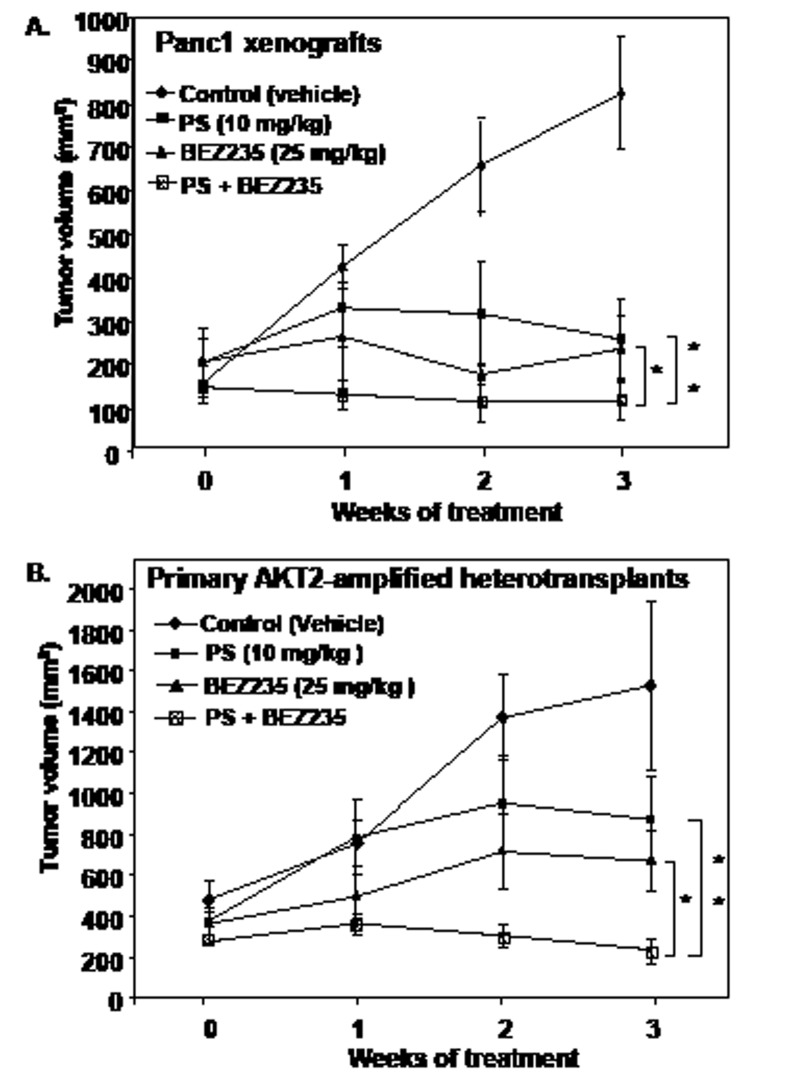
Co-treatment with PS and BEZ235 significantly inhibits tumor growth of cultured and primary AKT-amplified human pancreatic cancer heterotransplants A. Panc1 cells (5 × 10^6^) were injected into the flank of female athymic nude mice. Treatment was initiated when mean tumor volumes were approximately 100-200 mm^3^. Vehicle (5% DMSO, i.p.), PS (10 mg/kg t.i.w., i.p.), BEZ235 (25 mg/kg, daily by oral gavage) and a combination of BEZ235 and PS were administered for three weeks. Values represent mean tumor volume + S.E.M for all groups. * indicates values significantly less in the combination compared to treatment with BEZ235 alone (p < 0.01 as determined by a two-tailed, paired t-test). ** indicates values significantly less in the combination compared to treatment with PS alone (p < 0.01 as determined by a two-tailed, paired t-test). B. Primary AKT2-amplified human pancreatic cancer tumors were implanted subcutaneously into the flank of female athymic nude mice. Mice were randomized and treatment was initiated when the mean tumor volume reached 400 mm^3^. Vehicle (5% DMSO, i.p.), PS (10 mg/kg t.i.w., i.p.), BEZ235 (25 mg/kg, daily by oral gavage) and a combination of BEZ235 and PS were administered for three weeks. Values represent mean tumor volume + S.E.M for all groups. * indicates values significantly less in the combination compared to treatment with BEZ235 alone (p<0.05 as determined by a two-tailed, paired t-test). ** indicates values significantly less in the combination compared to treatment with PS alone (p<0.05).

In vivo anti-tumor activity of BEZ235 and/or PS against PDAC xenografts in mice. Next, we determined the effect of treatment with BEZ235 and/or PS on the growth of established (> 100 mm^3^) tumor xenografts of Panc1 cells subcutaneously implanted in the flanks of nude mice. As shown in Figure 5A, compared to control treatment (vehicle alone), a three week treatment with BEZ235 or PS alone significantly reduced tumor growth, resulting in lower mean tumor volume of the Panc1 tumors (p < 0.01). Notably, combined treatment with BEZ235 and PS completely abrogated the growth of Panc1 tumor xenografts, an effect significantly superior to treatment with BEZ235 or PS as a single agent (p< 0.05) (Figure 5A). We also determined the anti-tumor activity of BEZ235 and/or PS against tumor growth of subcutaneously implanted primary PDAC heterotransplants (possessing K-RAS mutation and AKT2 amplification) in the nude mice. As shown in Figure 5B, treatment with either BEZ235 or PS significantly reduced tumor growth of the heterotransplanted PDAC tumors (p < 0.01). BEZ235 exerted a slightly superior, but not statistically significant, anti-tumor effect on the growth of the heterotransplants in the nude mice (p > 0.05). Importantly, co-treatment with the two agents was significantly more effective in reducing the growth of the heterotransplanted tumors, resulting in lower mean tumor volume, as compared to treatment with either BEZ235 or PS (Figure 5B) (p < 0.05). At the doses that were used in both models, neither single agent nor the combination of BEZ235 and PS caused weight loss or other toxicity to the mice.

## DISCUSSION

**Figure 6:**
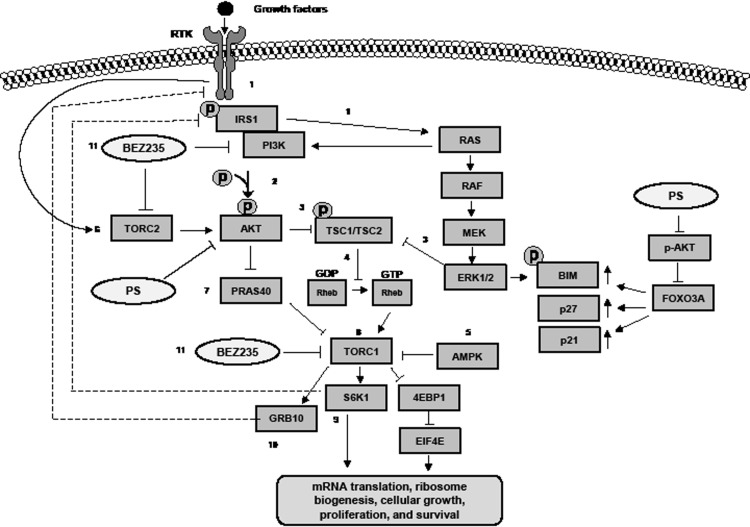
Activity of BEZ235 and PS against the mTOR signaling pathway in pancreatic cancer cells (1), Upon growth factor binding to a receptor tyrosine kinase (RTK), insulin receptor substrate (IRS1) is activated. This stimulates the PI3K and RAS signaling pathways. (2), Activation of PI3K and RAS results in phosphorylation and increased activity of the effector kinases, AKT and ERK1/2. (3), Activated AKT and ERK directly phosphorylate and inactivate the tuberous sclerosis (TSC) 1 /TSC2 complex and thus activate mTORC1. (4), When the TSC1/ TSC2 complex is active, it negatively regulates mTORC1 by converting RAS homolog enriched in brain (RHEB) from its active GTP-bound state to its inactive GDP-bound state. (5), Similar to AKT, the adenosine monophosphate-activated protein kinase (AMPK) can directly block the activity of TORC1 by phosphorylation of Raptor, a TORC1 inhibitor, leading to allosteric inhibition of TORC1. (6), The activated RTK can also directly activate TORC2 which phosphorylates AKT at Ser473 leading to inactivation of the TSC1/TSC2 complex and activation of TORC1. (7), Activated AKT also signals TORC1 by phosphorylating and inactivating PRAS40. Inactivating phosphorylation causes PRAS40 to disassociate from Raptor which releases inhibition on TORC1. (8), Activation of TORC1 leads to phosphorylation of 4EBP1 and S6K1 which in turn promote protein synthesis, ribosome biogenesis, cellular growth, proliferation and survival. (9), When activated by TORC1, S6K1 directly phosphorylates IRS1 leading to degradation of IRS and dampening of growth factor signaling. (10), In addition, growth factor receptor-bound protein 10 (GRB10) another substrate activated by TORC1 activity reduces the expression of the growth factor receptors (RTKs). (11), Treatment with BEZ235 inhibits the activity of PI3K, TORC1, and TORC2 resulting in depletion of p-AKT, p-4EBP1, p-p70S6K and cap-dependent translation with concomitant induction of BIM, p27 and p21. This leads to cell cycle growth inhibition and decreased cell proliferation of pancreatic cancer cells. PS treatment modestly inhibits TORC1 activity leading to depletion of p-4EBP1 and p-p70S6K. PS treatment also inhibits p-AKT leading to nuclear localization of FOXO3A, which induces expression of p21, p27, and BIM. Combined treatment with BEZ235 and PS causes greater induction of BIM and increased cell death of pancreatic cancer cells.

A profiling of the genetic alterations in human PDAC has shown that simultaneously increased activity of RAS-RAF-MEK-ERK1/2 and PI3K-AKT-mTOR pathways is common [2, 3]. The two pathways interact to regulate each other, and also co-regulate important downstream targets (Figure 6) [4]. This promotes not only the growth, survival and aggressive phenotype of PDAC cells, but also confers therapeutic resistance to chemotherapeutic agents or even single pathway-targeted agents [2, 4, 33, 39]. Consistent with this, previous studies have shown that although there is modest single agent activity of PI3K-AKT or MEK-ERK1/2 inhibitor, neither exhibits a meaningful in vivo tumor regression nor improves survival in PDAC [40-42]. Recently, the therapeutic role of targeting mTOR has also been evaluated in vivo against PDAC xenografts with increased activity of the PI3K-AKT-mTOR pathway from patients with advanced PDAC [19, 43]. Although some xenografts showed tumor regression, in the clinic, only 13% of patients exhibited stable disease and none experienced an objective response [19]. In the present studies we show that treatment with the dual PI3K-mTOR inhibitor BEZ235 and pan-histone deacetylase inhibitor PS, when administered alone, exert in vitro and in vivo antitumor activity against PDAC cells. These findings confirm other recent reports that have documented the preclinical single agent activity of PS or BEZ235 against PDAC cells [29, 37]. Importantly, our findings show, for the first time, that co-treatment with the two agents synergistically induces apoptosis and causes in vivo tumor regression of Panc1 and freshly harvested human PDAC implanted subcutaneously in the nude mice.

Our results also show that treatment with PS and BEZ235 induced the expression of p21^waf^ and p27^kip1^ which was associated with cell cycle growth arrest of PDAC cells. This is important because loss of function of p16 and ARF, which in turn can cause loss of p53 function, is frequent in and promotes cell cycle progression and growth of PDAC cells [2, 3]. The PDAC cells used here (Panc1 and Hs766T) are also known to possess genetic alterations leading to loss of function of p16 and p53 [44]. Consistent with previous reports, our findings also show that treatment with BEZ235 alone induced cell cycle growth arrest. This was associated with marked attenuation of TORC1 and TORC2 activity. Treatment with BEZ235 alone was modestly effective in inducing apoptosis of PDAC cells, which was associated with reduced levels of p-AKT and modest induction of BIM in the PDAC cells [29, 30]. In contrast, treatment with PS alone induced BIM and BAK, while simultaneously reducing MCL-1 and Bcl-x_L_ levels in the PDAC cells. Collectively, these effects of PS would lower the threshold for apoptosis and provide an explanation for why co-treatment with PS significantly enhanced BEZ235-induced apoptosis of the PDAC cells. Recent reports have highlighted superior anti-tumor activity of the combinations of dual PI3K-AKT-mTOR inhibitors with MEK-ERK1/2 inhibitors against tumors that express mutant K-RAS and exhibit increased activity of PI3K-AKT-mTOR pathway (Figure 6) [31, 45]. In present study, treatment with PS attenuated p-ERK1/2 levels (Supplemental Figure 2), thereby effectively functioning as an inhibitor of MEK-ERK1/2. It is well documented that ERK1/2 activity phosphorylates and reduces BIM levels, and inhibition of ERK1/2 induces BIM and promotes apoptosis (Figure 6) [46]. In the present studies, inhibition of AKT following co-treatment with BEZ235 and PS may also be promoting BIM induction by inhibiting phosphorylation of FOXO3A, thereby increasing FOXO3A levels in the nucleus leading to transcriptional up regulation of BIM and p27 (Figure 6) [47]. Induction of p27 due to PS treatment may also explain synergistic anti-tumor effects of co-treatment with PS and BEZ235; since a previous report has documented that the levels of p27 positively correlate with and sensitize cancer cells to BEZ235 treatment [48].

Co-treatment with BEZ235 and PS also caused marked reduction in p-AKT (Ser473) levels, abolishing the feedback activation of PI3K-AKT due to TORC1 inhibition, while maintaining marked attenuation of p-S6K1 (Thr389) and p-4EBP1 (Thr37/46) levels caused by depletion of TORC1 activity (Figure 6). TORC1-mediated phosphorylation of 4EBP1, and the resulting increased activity of eIF4E, appears to be a key regulator of proliferation and survival of transformed cells by causing up regulation of Myc, cyclin D1 and MCL-1 (Figure 6) [9]. Consequently, marked depletion of p-4EBP1 (Thr37/46) levels by combined treatment with PS and BEZ235 would inhibit growth and survival of PDAC cells, even of cells expressing mutant K-RAS and high PI3K-AKT-mTOR activity. Conversely, it is conceivable that *MYC* and *eIF4E* amplification in tumors may confer resistance against a BEZ235-based combination therapy [49].

Notably, a previous report showed that expression of non-phosphorylatable, constitutively active 4EBP1 suppressed growth of tumors driven by mutations in both PI3K and K-RAS [16]. This explains significantly greater tumor growth delay in two separate in vivo PDAC models utilized in present studies, i.e., the subcutaneously implanted Panc1 xenografts and PDAC heterotransplants in the nude mice [50]. It should also be noted that inhibition of TORC1 activity as well as treatment with PS has been shown to induce autophagy [43, 51]. In tumors expressing mutant RAS, autophagy has been shown to serve as a pro-death mechanism, such that RAS-induced autophagy led to caspase-independent cell death [52, 53]. Therefore, it is conceivable, although not specifically evaluated here, that co-treatment with BEZ235 and PS may lead to accentuated tumor cell stress and autophagy, leading to the engagement of an autophagic cell death mechanism and tumor growth inhibition. Furthermore, TORC1 inhibition by BEZ235 treatment and PS has been shown to inhibit tumor angiogenesis [29, 54]. Again, co-treatment with BEZ235 and PS may accentuate this mechanism and explain the superior in vivo antitumor activity of the combination in our study. It is clear that in the future more in depth studies would have to be performed to determine the pharmacodynamics correlates of the superior in vivo activity of this combination. Recently, co-treatment with an ATP competitive, dual TORC1 and TORC2 inhibitor was shown to enhance the pre-clinical anti-tumor efficacy of a pan-HDAC inhibitor against primary xenograft models of hepatocellular carcinoma [38]. Our preclinical in vivo findings presented here support a compelling rationale for further evaluating the combination of the dual PI3K-mTOR kinase inhibitor BEZ235 and PS in the in vivo models of human PDAC that express mutant K-RAS and increased activity of PI3K-AKT-mTOR axis.

## MATERIALS AND METHODS

### Cell culture

Human pancreatic cancer cell lines Panc1, Hs766T, MIA PaCa-2 and AsPC-1 were obtained from American Type Culture Collection (Manassas, VA). Cells were thawed and cultured for 3-5 passages, then frozen in aliquots in liquid nitrogen. All experiments with cell lines were performed within 6 months after thawing or obtaining from ATCC. Cell line characterization was performed by ATCC. The ATCC utilizes short tandem repeat (STR) profiling for characterization of cell lines. Panc1 and Hs766T cells were maintained in DMEM with 10% fetal bovine serum (FBS) and 1% penicillin streptomycin. MIA PaCa-2 cells were cultured in DMEM with 10% FBS, 2.5% horse serum and 1% penicillin streptomycin. AsPC-1 cells were cultured in RPMI with 10% FBS and 1% penicillin streptomycin. Cells were passaged 2-3 times per week. Logarithmically growing cells were used for all experiments detailed below. Cells were washed free of the drugs prior to harvesting for experimentation.

### Reagents and antibodies

Panobinostat (PS) and BEZ235 (Supplemental Figure S1: chemical structures of BEZ235 and PS) were kindly provided by Novartis Pharmaceuticals, Inc. Anti-p-AKT (Ser473), anti-AKT, anti-p-4EBP1(Thr37/46), anti-4EBP1, anti-p-p70S6K (Thr389), anti-p70S6K, anti-p-ERK1/2, anti-ERK1/2, anti-BAK and anti-BIM antibodies were purchased from Cell Signaling Technology. Anti-acetylated histone H3 antibody was obtained from Millipore. Anti-Bcl-x_L_, anti-BAX, anti-c-Myc, anti-MCL-1 and anti-p21^waf^ were obtained from Santa Cruz Biotechnology, Inc. Anti-p27^kip1^ antibody was obtained from BD Transduction Labs. Anti-β-actin and anti-acetylated α-tubulin antibodies were obtained from Sigma-Aldrich.

### Cell viability

Cell viability was assayed using a Cell Counting Kit-8 (Dojindo Molecular Technologies, Inc.) according to the manufacturer's protocol. Briefly, cells were treated with the indicated concentrations of PS and BEZ235 for 48 hours. Water soluble tetrazolium salt (WST-8) was added and incubated at 37°C for 2 hours and the absorbance was measured at 450 nm. The percentages of viable cells are expressed relative to the viability of the untreated control cells.

### Cell cycle analysis

The flow cytometric evaluation of cell-cycle status and the percentage of cells in the G_1_, S, and G_2_–M phases was performed according to a previously described method [55]. Briefly, after the designated treatments, cells were harvested and washed twice with 1X phosphate-buffered saline (PBS), then fixed in 70% ethanol overnight at −20°C. The cells were washed twice with 1X PBS, incubated with RNase A and propidium iodide for 15 min at 37°C in the dark. Cell cycle data were collected on a BD Accuri C6 flow cytometer (BD Biosciences) with a 488 nm laser and analyzed as previously described [55].

### Cell lysis and protein quantitation

Untreated or drug-treated cells were centrifuged, washed with 1X PBS and the cell pellets were re-suspended in 200 μL lysis buffer and incubated on ice for 30 minutes, as previously described [32, 55]. After centrifugation, the protein concentration was quantified using a BCA reagent (Pierce), according to the manufacturer's protocol.

### SDS-PAGE and Western blotting

Fifty micrograms of total cell lysate was used for SDS–polyacrylamide gel electrophoresis (PAGE). Western blot analyses were performed on total cell lysates using specific antisera or monoclonal antibodies as previously described [55]. After thorough washing in 1X PBST, blots were incubated in IRDye 680 goat anti-mouse or IRDye 800 goat anti-rabbit secondary antibodies (LI-COR) for 1 hour, washed 3 times in 1X PBST, and scanned with an Odyssey Infrared Imaging System (LI-COR). The expression levels of β-actin were used as the loading control for the immunoblots. Immunoblot analyses were performed at least twice and representative blots are presented. Densitometric evaluation of proteins was performed with Image Quant 5.2 software.

### Assessment of apoptosis and isobologram analyses

After drug treatments, cells were washed with 1X PBS, resuspended in 100 μL of annexin V staining solution (containing annexin V-FITC conjugate (BD Biosciences) and TO-PRO-3 iodide in a HEPES buffer). Following incubation at room temperature for 15 minutes, cells were analyzed on an Accuri C6 flow cytometer (BD Biosciences). Synergism defined as more than expected additive effect was assessed using the median dose effect equation of Chou-Talalay (assuming mutual exclusivity) [56]. The combination index (CI) for each drug combination was obtained using the commercially available software Calcusyn (Biosoft, Ferguson, MO). CI < 1, CI = 1, and CI > 1 represent synergistic, additive or antagonistic interaction of the two agents, respectively.

### In vivo animal studies

Six-week-old female nude mice were purchased from the Harlan Laboratories, Inc. and were maintained under specific pathogen-free conditions. All animal experiments were approved by the IACUC at the Kansas University Medical Center and adhered to the NIH *Policy on Humane Care and Use of Laboratory Animals*. For Panc1 xenografts 5 × 10^6^ Panc1 cells were injected subcutaneously into the right flank of the mice. When the mean tumor volume reached approximately 100-200 mm^3^, mice were randomized into one of four treatment groups: (a) vehicle (5% DMSO), (b) PS (10 mg/kg, i.p. three times per week), (c) BEZ235 (25 mg/kg, daily by oral gavage), (d) combination of PS (10 mg/kg) and BEZ235 (25 mg/kg). PS was prepared fresh in 5% DMSO. BEZ235 was prepared fresh in 10% NMP/PEG 300 [1-methyl-2-pyrrolidone/polyethylene glycol 300; 10:90, v/v]. General condition of the mice was monitored daily. Tumors were measured by digital calipers every two days and tumor volume was calculated using the formula (length × (width^2^)/2. AKT-amplified human pancreatic cancer heterotransplants were kindly provided by Manuel Hidalgo and Anirban Maitra (Johns Hopkins University) [19, 30]. Tumor sections (3 × 3 mm) were implanted into the flank of athymic nude mice and allowed to grow to 1500 mm^3^. Mice were humanely sacrificed and the tumors were aseptically harvested, divided, and transplanted into the flank of an additional 24 mice. Tumors were allowed to grow until they reached approximately 200-400 mm^3^. The mice were then randomized into one of four treatment groups: (a) vehicle (5% DMSO), (b) PS (10 mg/kg i.p. three times per week), (c) BEZ235 (25 mg/kg, daily by oral gavage), (d) combination of PS (10 mg/kg) and BEZ235 (25 mg/kg). General condition of the mice was monitored daily. Tumor volumes were determined using digital calipers. Measurements were obtained every two days and tumor volume was calculated using (length × (width^2^)/2. Growth curves for tumors were plotted as the mean tumor volume + SEM from each treatment group.

### Statistical analysis

Significant differences between values obtained in a population of control and treated pancreatic cancer cells were determined using the Student's t test. P values less than 0.05 were assigned significance. For the in vivo studies, significant differences in the mean tumor volumes after three weeks of treatment with BEZ235 and/or PS were determined using a two-tailed, paired t-test.

## Supplementary Figures


