# Supplementary material for: Superior efficacy of co-treatment with dual PI3K/mTOR inhibitor NVP-BEZ235 and pan-histone deacetylase inhibitor against human pancreatic cancer
Source: Oncotarget. 2012 Nov 15;3(11):1416–27. doi: 10.18632/oncotarget.724 (PMC3717802; doi:10.18632/oncotarget.724)
Supplement: Supplementary file 1 [file oncotarget-03-1416-s001.pdf]

Superior efficacy of co-treatment with dual PI3K/mTOR inhibitor NVP-BEZ235 and pan-histone deacetylase inhibitor against human pancreatic cancer - Venkannagiri et al

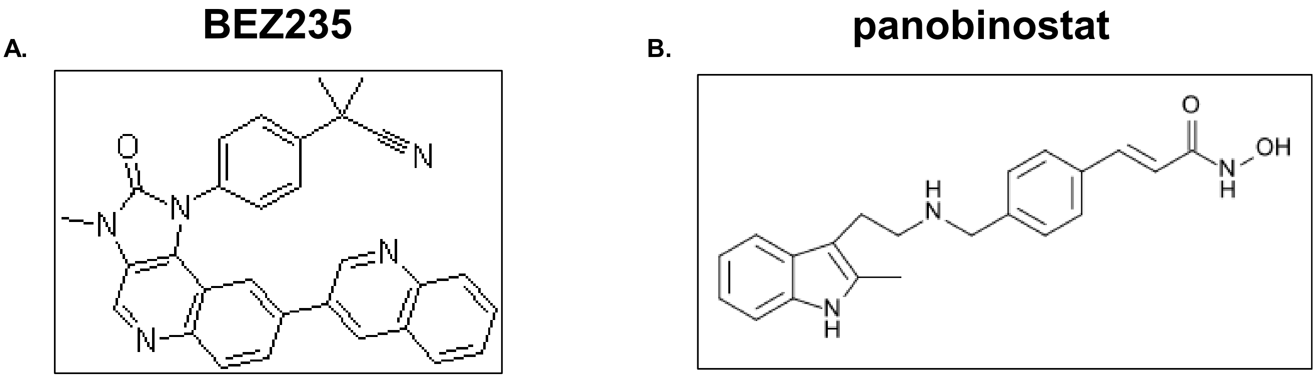

Supplemental Figure S1: Chemical structures for BEZ235 and panobinostat.

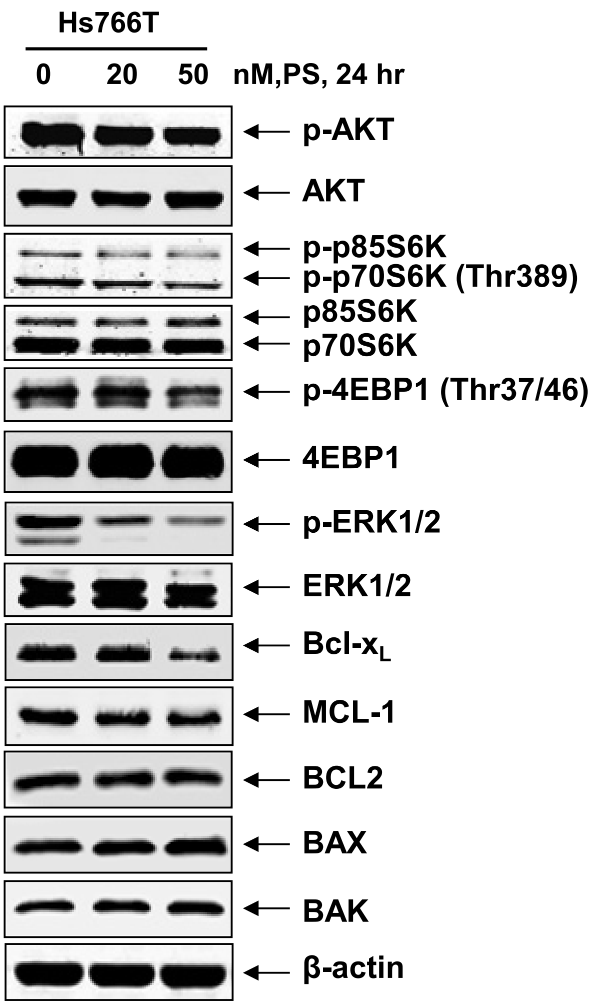

Supplemental Figure S2: Effects of treatment with PS on mTOR signaling and BCL2 family proteins in pancreatic cancer cells. Hs766T cells were treated with the indicated concentrations of PS for 24 hours. After treatment, total cell lysates were prepared and immunoblot analyses were performed for p-AKT (S473), AKT, p-p70S6K, p70S6K, p-4EBP1, 4EBP1, p-ERK1/2, ERK1/2, Bcl-xL, MCL-1, BCL2, BAX, and BAK. The expression levels of β-actin in the lysates served as the loading control.
